# Lactate Clearance as a Prognostic Marker in Severe Trauma: A Retrospective Cohort Study

**DOI:** 10.3390/diagnostics16152463

**Published:** 2026-08-05

**Authors:** Lotfi Rebai, Melinda Sammary, Olfa Faten, Sabrine Ben Brahem, Firas Kalai, Ichraf Ardhaoui, Lamia Thabet

**Affiliations:** 1 Department of Anesthesiology and Critical Care, Traumatology and Major Burns Center of Ben Arous, Faculty of Medicine of Tunis, University of Tunis El Manar, Tunis 1006, Tunisia; sammarymelinda@gmail.com (M.S.); olfa.faten@fmt.utm.tn (O.F.); sabrinaben1405@gmail.com (S.B.B.); kalaifiras28@gmail.com (F.K.); ichrafardhaoui2014@gmail.com (I.A.); 2 Laboratory of Microbiology, Traumatology and Major Burns Center of Ben Arous, Faculty of Medicine of Tunis, University of Tunis El Manar, Tunis 1006, Tunisia; thabetlamia@gmail.com

**Keywords:** lactate clearance, lactate kinetics, severe trauma, ICU, prognostic marker, in-hospital mortality

## Abstract

**Background/Objectives:** Lactate is a key biomarker of tissue hypoperfusion in severe trauma. While admission lactate is widely used, the prognostic value of delayed lactate clearance remains a subject of ongoing investigation, notably due to the inherent risk of survivorship bias. This study assessed the association between lactate kinetics and in-hospital mortality in a cohort of severely injured patients admitted to the intensive care unit. **Methods:** We conducted a single-center retrospective cohort study including adult patients (≥18 years) admitted to the ICU for severe trauma between January 2018 and December 2022. Arterial lactate was measured at H0, H4 (±1 h), and H12 (±1 h). Lactate clearance (LC) was calculated using the formula: LC (%/h) = [(Lactate_t1 − Lactate_t2)/Lactate_t1] × (1/Δt) × 100, where positive values indicate decreasing lactate (clearance) and negative values indicate worsening lactatemia. LC was calculated over H0–H4, H0–H12, and H4–H12. Among the 38 patients who died before 12 h and were excluded from the LC H0–H12 analysis, non-survivors were significantly older, had higher severity scores, and presented more frequently with hemorrhagic shock. Predictive performance was evaluated using ROC curves and multivariate logistic regression. The composite prognostic score was assessed using bootstrap internal validation (1000 resamples) to provide an optimism-adjusted AUC estimate. **Results:** Among 318 patients (median age 36 years; 86.5% male), hyperlactatemia (>2.2 mmol/L) was present in 70.1% at admission and persisted in 39.9% at 12 h. Non-survivors exhibited higher lactate levels and lower LC at all time points. LC H0–H12 demonstrated the best predictive performance for in-hospital mortality (AUC = 0.75; 95% CI [0.69–0.81]; *p* < 0.001). For early mortality (≤48 h), LC H0–H12 achieved an AUC of 0.80 (95% CI [0.71–0.89]; *p* < 0.001). A composite prognostic score incorporating age >60 years, GCS ≤ 7, prothrombin time ≤ 55%, pH ≤ 7.29, and LC H0–H12 > −2.93 %/h demonstrated good discrimination (AUC = 0.84; optimism-adjusted AUC = 0.82). **Conclusions:** Lactate levels and 12 h lactate clearance are valuable prognostic markers in severe trauma. Given the inherent survivorship bias affecting the LC H0–H12 analysis, its prognostic performance should be interpreted with appropriate caution and within a multimodal clinical assessment. The proposed composite score is promising but requires prospective external validation before clinical implementation.

## 1. Introduction

Lactate is a key metabolic biomarker traditionally associated with tissue hypoperfusion and cellular hypoxia during circulatory failure. It has long been interpreted as a marker of anaerobic metabolism and cellular distress [1,2]. Persistent hyperlactatemia is consistently associated with poor outcomes in critically ill patients, whereas a decrease in lactate levels during resuscitation reflects improved tissue perfusion and has been linked to better survival [3,4]. For these reasons, lactate monitoring is widely recommended in intensive care settings and has been incorporated into the definition of septic shock [5].

However, lactate is no longer considered merely a byproduct of anaerobic glycolysis. It is now recognized as a complex marker of metabolic stress and impaired cellular energetics, reflecting the interplay between oxygen delivery, mitochondrial function, adrenergic stimulation, and microcirculatory alterations [6]. This broader understanding is particularly relevant in trauma, where hyperlactatemia may persist despite apparent hemodynamic stabilization, indicating ongoing occult hypoperfusion or cellular dysfunction [7].

Severe trauma represents a major global health burden and remains a leading cause of death, particularly among young adults [8]. Early identification of patients at high risk of deterioration is crucial to optimize resuscitation strategies. In this context, lactate has emerged as a valuable diagnostic and prognostic biomarker. Admission lactate has been shown to correlate with injury severity and mortality, even in patients with initially normal vital signs [9].

More recently, lactate kinetics, particularly lactate clearance, have been proposed as dynamic indicators of resuscitation adequacy. Several studies have demonstrated an association between impaired lactate clearance and increased mortality in trauma patients. Large observational cohorts and systematic reviews, including the work by Dezman et al., have confirmed the prognostic value of both initial lactate and lactate clearance in trauma populations [10]. In addition, randomized trials such as the RePHILL study have incorporated lactate clearance into composite clinical endpoints, further supporting its clinical relevance, although without consistently demonstrating a direct impact on mortality [11].

Despite this growing body of evidence, the clinical utility of delayed lactate clearance remains controversial. In particular, lactate clearance measured at later time points is subject to survivorship bias, as only patients who survive the early phase of trauma are available for evaluation at 12 h.

Therefore, the present study aimed to evaluate the prognostic significance of lactatemia and lactate clearance in severely injured patients admitted to the intensive care unit. Specifically, we sought to determine:

(1) Whether lactate levels at different time points are associated with in-hospital mortality,
(2) Whether lactate clearance provides additional prognostic value compared with static lactate measurements.

## 2. Materials and Methods

### 2.1. Study Design and Setting

This retrospective observational study was conducted in the Department of Anesthesiology and Critical Care between January 2018 and December 2022. The study protocol was approved by the local ethics committee (Ethics Committee of the CTMB, 2024-RCS-P1, 20 January 2024). Due to the retrospective nature of the data collection, the requirement for individual informed consent was waived. All patients admitted to the ICU during the study period were managed according to standardized trauma resuscitation protocols consistent with ATLS guidelines and institutional damage control resuscitation principles, including balanced blood product administration, early hemorrhage control, and permissive hypotension where clinically appropriate. Variations in specific therapeutic decisions occurred within this consistent institutional framework at the discretion of the attending physician.

### 2.2. Study Population

Adult patients (≥18 years) admitted to the intensive care unit (ICU) for severe trauma were eligible. Severe trauma was defined according to the Vittel criteria [12] and/or the presence of life-threatening conditions at admission. Inclusion criteria included ICU admission for severe trauma and availability of arterial lactate measurements at admission (H0), 4 h (H4 ±1 h), and 12 h (H12 ±1 h). Exclusion criteria included missing key data, particularly lactate measurements, transfer to another facility within 24 h, or absence of initial trauma assessment at admission. It should be noted that patients who died before 12 h from admission (*n* = 38) could not contribute to the LC H0–H12 analysis. This constitutes an inherent survivorship bias, as this subgroup necessarily included the most severely ill individuals. The demographic and clinical characteristics of these 38 early deaths are described in Section Characteristics of Patients Who Died Before 12 Hours to allow the reader to contextualize the potential impact of this exclusion on the prognostic performance attributed to LC H0–H12. In addition, a comparison between the 41 patients excluded due to missing data and the 318 patients included in the final analysis is provided in the Section 3.

### 2.3. Data Collection

Data were extracted from electronic medical records using a standardized collection form. Collected variables included demographics, including age, sex, comorbidities, and ASA classification [13], trauma characteristics, clinical variables at admission such as vital signs and Glasgow Coma Scale (GCS), severity scores including RTS, ISS, TRISS, MGAP, and SAPS II, biological parameters such as arterial lactate levels, and therapeutic variables including use of vasopressors, mechanical ventilation, and transfusion. Blood transfusion was further categorized as red blood cell (RBC) units transfused within the first 24 h, and massive transfusion was defined as ≥10 units of RBC within 24 h. All data were collected at admission, ICU admission, H4, and H12.

### 2.4. Definitions

The primary endpoint was in-hospital mortality. Secondary endpoints included hemorrhagic shock, defined as ≥4 units of RBC within 6 h and/or persistent hypotension requiring vasopressors, nosocomial infection, and severe trauma defined as ISS >15. Early mortality was defined as death occurring within 48 h of admission.

### 2.5. Lactate Measurement and Lactate Kinetics

Arterial lactate levels were obtained from arterial blood gas analyses at H0, H4 (±1 h), and H12 (±1 h). It is important to note that these measurements were performed after ICU admission, at which point initial resuscitation had generally already been initiated in the emergency department. Lactate clearance (LC) was calculated according to the formula described by Régnier et al. [14]:

LC (%/h) = [(Lactate_t1 − Lactate_t2)/Lactate_t1] × (1/Δt) × 100

where Lactate_t1 denotes the lactate level at the earlier time point, Lactate_t2 the lactate level at the later time point, and Δt the time interval in hours. A positive LC value indicates a decrease in lactate (clearance), reflecting improved metabolic status. A negative LC value indicates an increase in circulating lactate (worsening lactatemia), reflecting ongoing or worsening tissue hypoperfusion. LC was calculated over three intervals: H0–H4, H0–H12, and H4–H12. The term “lactate kinetics” is used interchangeably with “lactate clearance” throughout this manuscript, as the formula reflects the net change in circulating lactate levels rather than true metabolic hepatic clearance.

### 2.6. Statistical Analysis

Statistical analyses were performed using SPSS version 26.0. A *p*-value < 0.05 was considered statistically significant. Normality was assessed using the Kolmogorov–Smirnov test. Continuous variables were compared using Student’s *t*-test or Mann–Whitney U test; categorical variables using Chi-square or Fisher’s exact test. ROC curves were constructed to evaluate predictive performance, with optimal thresholds determined using the Youden index. Confidence intervals for all reported AUC values are provided at the 95% level. Multivariate logistic regression was performed to identify independent predictors of in-hospital mortality, with variables with *p* < 0.1 in univariate analysis included in the model. To address the risk of optimism bias inherent in deriving and evaluating the composite prognostic score on the same dataset, bootstrap internal validation was performed with 1000 resamples, and the resulting optimism-adjusted AUC is reported alongside the apparent AUC. LC values were also tested in combination with established trauma scores (RTS, ISS, TRISS, MGAP, SAPS II) to assess their incremental predictive contribution.

## 3. Results

### 3.1. Study Population

**Figure 1:**
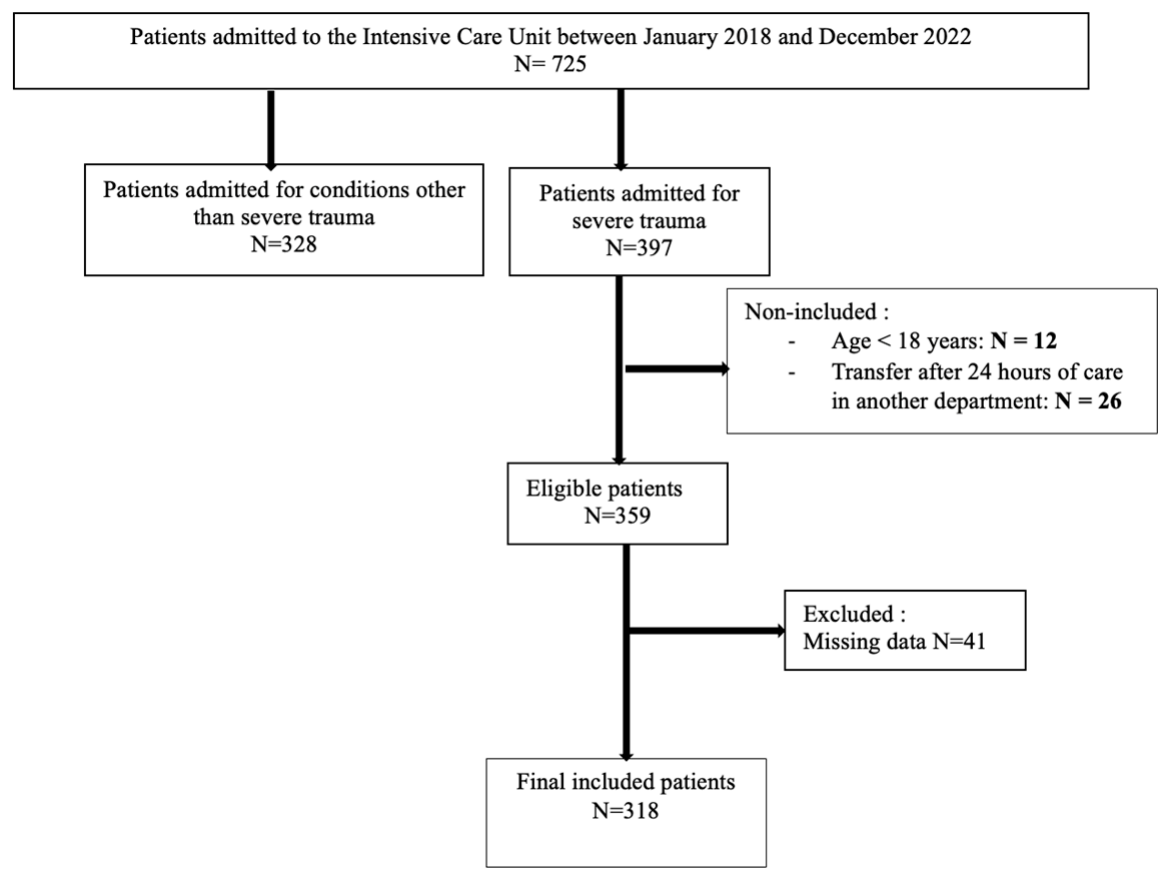
Flow chart of the study.

During the study period, 725 patients were admitted to the ICU, of whom 397 were admitted for severe trauma. After applying inclusion and exclusion criteria, 318 patients were included in the final analysis (Figure 1). Among the 41 patients excluded due to missing data, available records showed a tendency towards greater physiological derangement at admission (lower mean GCS, higher median SAPS II), suggesting that this subgroup may have been more severely ill than the included population. While this proportion represents 10.3% of eligible patients, a residual selection bias cannot be entirely excluded and is acknowledged as a limitation. The median age was 36 years (IQR: 27–54), and the majority of patients were male (86.5%, *n* = 275). The overall in-hospital mortality rate was 33.6% (*n* = 107).

#### Characteristics of Patients Who Died Before 12 Hours

Among the 397 patients admitted for severe trauma, 38 died within the first 12 h of ICU admission and were therefore excluded from the LC H0–H12 analysis. Compared with the 318 patients included in the final cohort, these early non-survivors were significantly older (median age 47 vs. 36 years, *p* = 0.003), had higher SAPS II scores (median 61 vs. 34, *p* < 0.001), more frequently presented with hemorrhagic shock (68.4% vs. 24.2%, *p* < 0.001), and had markedly elevated admission lactate levels (median 9.8 vs. 3.9 mmol/L, *p* < 0.001) (Table 1). These findings confirm that early deaths represent a distinct and more severely ill subgroup. Their exclusion from the LC H0–H12 analysis inevitably introduces an upward bias in the apparent discriminative performance of this parameter, and the reported AUC of 0.75 for in-hospital mortality should be interpreted in this context.

**Table 1:** Demographic data and clinical characteristics of polytrauma patients.

Characteristics	Survivors (*n* = 211)	Deceased (*n* = 107)	*p*-Value
Sex:			
Male	183 (86.7%)	92 (86%)	0.854
Female	28 (13.3%)	15 (14%)	
Age (years)	35 (25; 50)	42 (30; 61)	0.001
ASA score:			
ASA I	167 (79.1%)	76 (71%)	0.105
ASA II	43 (20.4%)	28 (26.2%)	0.241
ASA III	1 (0.5%)	3 (2.8%)	0.078
Type of trauma:			
Penetrating	18 (8.5%)	7 (6.5%)	0.534
Blunt	193 (91.5%)	100 (93.5%)	
Circumstances:			
RTA (road traffic accident)	158 (74.9%)	83 (77.6%)	0.597
Fall	36 (17.1%)	21 (19.6%)	0.573
Blast effect	1 (0.5%)	0 (0%)	0.476
Stab wound	9 (4.2%)	1 (0.9%)	0.108
Other	7 (3.3%)	2 (1.9%)	0.462
ICU admission delay (hours)	3 (1.5; 5.5)	2 (1; 4)	0.006
Localisation of trauma			
Head	99 (46.9%)	84 (78.5%)	<0.001
Axial	59 (28%)	42 (39.3%)	0.041
Thorax	67 (31.8%)	48 (44.9%)	0.022
Abdomen	74 (35.1%)	41 (38.3%)	0.305
Pelvis	53 (25.1%)	37 (34.6%)	0.071
Vascular	11 (5.2%)	7 (6.5%)	0.628
Limb	64 (30.3%)	43 (40.2%)	0.079
Initial Clinical Examination:			
GCS	11 (7; 14)	6 (3; 11)	<0.001
Anisocoria and/or mydriasis	22 (10.4%)	38 (35.5%)	<0.001
Convulsion	6 (2.8%)	1 (0.9%)	0.273
Mean arterial pressure, mmHg	82.15 ± 20.45	78.67 ± 21.05	0.093
Heart rate	95.11 ± 21.95	98.39 ± 26.42	0.197
Massive hemorrhage	37 (17.5%)	40 (37.4%)	<0.001
Cardiac arrest	1 (0.5%)	5 (4.7%)	0.009
Peripheral oxygen saturation, %	93.38 ± 8.81	87.79 ± 13.22	<0.001
Severity Scores:			
MGAP	22 (19; 26)	17 (15; 22)	<0.001
SAPS II	28 (19.75; 39)	46 (35; 56)	<0.001
RTS	6.61 (5.96; 7.84)	5.03 (4.09; 6.37)	<0.001
ISS	21 (14; 29)	29 (22; 41)	<0.001
TRISS	5.4 (2; 17.6)	35 (12.2; 61.5)	<0.001
Biological Data:			
Hemoglobin (Hb, g/dL)	11.56 ± 2.30	10.72 ± 2.77	0.013
Hematocrit (Ht, %)	33.73 ± 6.49	31.76 ± 7.83	0.057
White blood cells (WBC,/mm^3^)	17,726.29 ± 6567.05	18,145.60 ± 6754.97	0.299
Platelets (Plq,/mm^3^)	202,578 ± 70,105	182,649 ± 63,204	0.014
Fibrinogen (g/L)	2.55 ± 1.29	1.94 ± 1.00	<0.001
Prothrombin time (PT, %)	72.85 ± 19.14	57.59 ± 22.59	<0.001
Urea (mmol/L)	4.9 (3.89; 5.95)	5.52 (4.15; 7.24)	0.003
Creatinine (µmol/L)	67 (56; 85)	81.5 (63; 117.25)	<0.001
ASAT (IU/L)	66.95(34.07;120.75)	67 (42.9; 121.5)	0.588
ALAT (IU/L)	42.2 (19; 73.57)	37.63 (21; 73.75)	0.709
Procalcitonin (ng/mL)	0.165 (0.05; 0.85)	0.14 (0.05; 1.27)	0.632
pH	7.38 ± 0.10	7.27 ± 0.18	<0.001
HCO_3_^−^ (mmol/L)	21.89 ± 4.33	17.88 ±4.57	<0.001
PaCO_2_ (mmHg)	34.57 ± 9.2	38.65 ± 13.90	0.047
Emergency procedures:			
Intubation and sedation (IOT)	129 (61.1%)	91 (85%)	<0.001
Osmotherapy	25 (11.8%)	37 (34.6%)	<0.001
Transfusion	73 (34.6%)	51 (47.7%)	0.024
Massive transfusion	12 (5.7%)	16 (15%)	0.006
Packed erythrocytes transfusion	3.60 ± 2.76	4.96 ± 3.74	0.007
Vasoactive drugs	116 (55%)	93 (86.9%)	<0.001
Fluid resuscitation (mL)	1948 ± 1596	2594 ± 2194	0.009
Tranexamic acid	37 (17.5%)	33 (30.8%)	0.007
Thoracic drainage	44 (20.9%)	22 (20.6%)	0.952
Emergency surgery	51 (24.2%)	24 (22.4%)	0.730
Outcome:			
Duration of ICU (days)	11 (5; 19)	5 (1; 12)	<0.001
Mechanical ventilation (days)	7 (0.25; 13)	5 (0.83; 12)	0.817
Nosocomial infection	99 (46.9%)	39 (36.4%)	0.075

### 3.2. Lactate Levels and Lactate Kinetics

At ICU admission (H0), 223 patients (70.1%) presented with hyperlactatemia >2.2 mmol/L. At H4, 187 patients (58.8%) remained hyperlactatemic. At H12, 127 patients (39.9%) remained hyperlactatemic. With regard to lactate kinetics, worsening lactatemia (negative LC exceeding 10 %/h in absolute value) was observed in 39 patients (12.3%) between H0 and H4, in 10 patients (3.1%) between H0 and H12, and in 7 patients (2.2%) between H4 and H12.

### 3.3. Comparison Between Survivors and Non-Survivors

Mean lactate levels at all time points were significantly higher in non-survivors: H0, 5.5 ± 4.3 vs. 3.3 ± 2.1 mmol/L; H4, 5.3 ± 4.4 vs. 2.8 ± 2.1 mmol/L; H12, 4.8 ± 4.6 vs. 2.0 ± 1.7 mmol/L (all *p* < 0.001). Lactate kinetics were significantly impaired in non-survivors: LC H0–H4, 0.6 (−4.4; 8.3) vs. −5.4 (−9.7; 0.9) %/h; LC H0–H12, −0.4 (−2.9; 3.6) vs. −3.5 (−5.2; −1.7) %/h; LC H4–H12, −0.8 (−4.6; 3.7) vs. −3.8 (−5.8; −1.3) %/h (all *p* < 0.001). In accordance with the formula used, negative LC values reflect an increase in circulating lactate over the corresponding time interval, indicating ongoing or worsening tissue hypoperfusion.

### 3.4. Mortality Analysis

**Figure 2:**
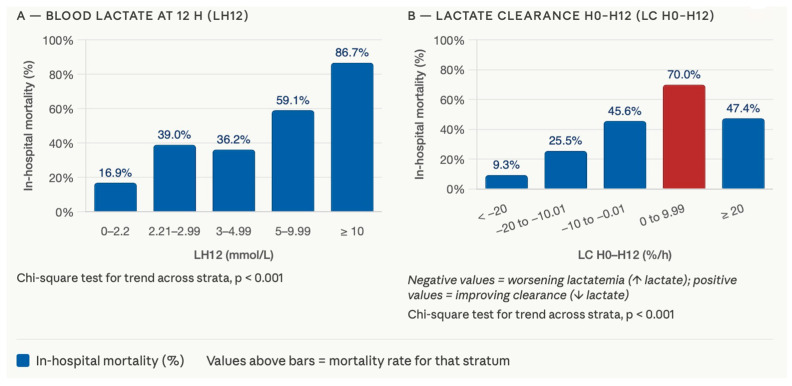
In-hospital mortality rates stratified by (**A**) blood lactate level at 12 h post-admission (LH12, mmol/L) and (**B**) hourly lactate clearance between admission and 12 h (LC H0–H12, %/h). Mortality was calculated within each stratum as the number of deaths divided by total patients in that group. Panel (**A**) shows a progressive increase in mortality with increasing LH12, with rates ranging from 16.9% in the lowest stratum (0–2.2 mmol/L) to 86.7% in the highest (≥10 mmol/L). Panel (**B**) demonstrates a non-linear relationship between LC H0–H12 and mortality, with the highest mortality (70.0%) in patients with LC H0–H12 between 0 and +9.99 %/h, and a plateau effect at extreme values. Sign convention (panel (**B**)): a negative LC H0–H12 value indicates a rise in circulating lactate over the H0–H12 interval (worsening lactatemia), while a positive value indicates a fall in lactate (clearance); the most negative stratum (<−20 %/h) corresponds to patients with the greatest improvement in lactate and was associated with the lowest mortality (9.3%). Between-group comparisons performed using chi-square test for trend; *p* < 0.001 for both panels. LC: lactate clearance; LH12: blood lactate at 12 h; %/h: percent per hour.

**Figure 3:**
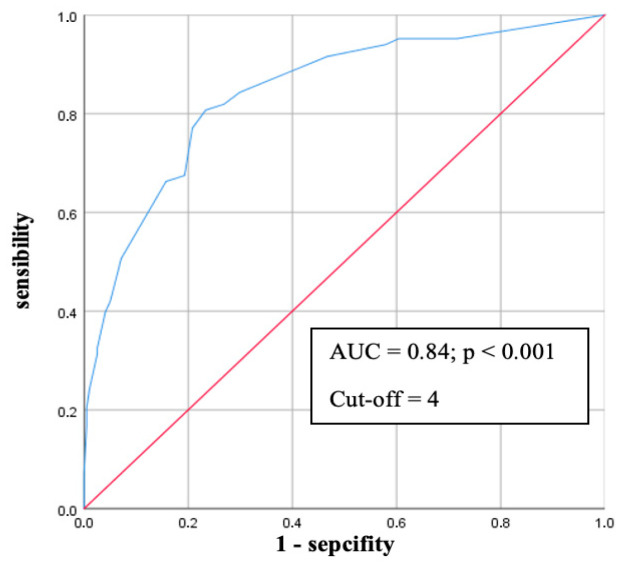
ROC curve of the predictive score for in-hospital mortality.

The early mortality rate (≤48 h) was 12.6%; in-hospital mortality was 33.6%. ROC curve analysis demonstrated that LC H0–H12 was the strongest predictor of in-hospital mortality (AUC = 0.75; 95% CI [0.69–0.81]; *p* < 0.001), and H12 lactate also showed good performance (AUC = 0.73; 95% CI [0.67–0.79]; *p* < 0.001). For early mortality, the best predictors were LC H0–H12 (AUC = 0.80; 95% CI [0.71–0.89]; *p* < 0.001) and H0 lactate (AUC = 0.75; 95% CI [0.64–0.85]; *p* < 0.001). These results, now clearly separated by outcome type, are summarized in Table 2. A progressive increase in mortality was observed with increasing lactate levels at all time points, while a plateau effect was noted in the relationship between lactate kinetics and mortality (Figure 2 and Figure 3).

### 3.5. Prediction of Secondary Outcomes

Lactate levels and lactate clearance were modest but significant predictors of severe trauma (ISS > 15), and lactate levels showed good predictive performance for massive hemorrhage. Neither lactate levels nor lactate clearance were associated with nosocomial infections.

### 3.6. Incremental Prognostic Value

Multivariate analysis demonstrated that both H0 and LC H0–H12 significantly improved the predictive performance of trauma severity scores (RTS, ISS, TRISS, MGAP), although H0 did not significantly improve the predictive value of SAPS II (*p* = 0.375). The incremental value analysis is presented in Table 3.

### 3.7. Development of a Prognostic Score

Five independent predictors of in-hospital mortality were identified: age > 60 years, GCS ≤ 7, prothrombin time ≤ 55%, pH ≤ 7.29, and LC H0–H12 > −2.93 %/h. A 10-point composite prognostic score was developed (Table 4 and Table 5), defining three risk categories: low risk (score ≤ 3, mortality < 10%), intermediate risk (score 4–6), and high risk (score ≥ 7, mortality > 90%). The score demonstrated good discriminative performance for in-hospital mortality, with an apparent AUC of 0.84 (95% CI [0.79–0.89]; *p* < 0.001). Following bootstrap internal validation (1000 resamples), the optimism-adjusted AUC was 0.82, confirming the robustness of the model’s discriminative ability within this derivation cohort. External prospective validation is required before clinical implementation.

## 4. Discussion

### 4.1. Principal Findings

This study demonstrates that both static lactate levels and lactate kinetics are significantly associated with mortality in severely injured patients. Among the parameters evaluated, lactate clearance between admission and 12 h (LC H0–H12) showed the best predictive performance for in-hospital mortality. Additionally, both initial lactate (H0) and LC H0–H12 improved the discriminative ability of commonly used trauma severity scores.

### 4.2. Mortality Rate and Case-Mix

The observed in-hospital mortality rate of 33.6% in our cohort was higher than that reported in most trauma registries. For comparison, a multicenter French study reported a 30-day mortality of 23% [15], whereas North American cohorts described rates as low as 9.2% in specialized trauma centers [16]. This discrepancy may be explained by differences in case-mix, including a higher proportion of patients with severe physiological derangement, delayed referral, or limited prehospital resources. It also likely reflects variations in trauma system organization and access to definitive care.

### 4.3. Prognostic Value of Initial Lactate

Consistent with prior studies, admission lactate was strongly associated with mortality. Several authors, including Odom and Parsikia, have shown significantly higher initial lactate levels in non-survivors [17,18]. Our findings confirm this relationship, with a clear gradient of increasing mortality across lactate strata. Importantly, these results highlight the absence of a universal lactate threshold in trauma. While our optimal cut-off of 4.78 mmol/L aligns with the findings of Régnier et al. [14], other studies have reported lower thresholds of 2–2.5 mmol/L, likely reflecting heterogeneity in patient populations and clinical settings [19]. These observations reinforce the view that lactate should be interpreted as a global indicator of metabolic stress rather than against a universal threshold, integrating tissue perfusion, adrenergic activation, and mitochondrial dysfunction.

### 4.4. Lactate Clearance: Strengths and Limitations

Lactate clearance was significantly impaired in non-survivors, supporting its role as a dynamic marker of resuscitation adequacy. These findings are in agreement with previous studies, including those by Abramson and Régnier [14,20], which reported delayed lactate normalization in patients with poor outcomes. In our study, LC H0–H12 demonstrated better predictive performance than early lactate clearance (H0–H4), particularly for early mortality.

However, as documented in Section Characteristics of Patients Who Died Before 12 Hours, 38 patients died before H12 and were excluded from this analysis. These early non-survivors were markedly more severely ill, and their exclusion introduces a systematic upward bias on the apparent AUC of LC H0–H12. The reported value of 0.75 for in-hospital mortality therefore likely overestimates the true discriminative performance of this parameter in an unselected trauma population, and should be interpreted with this important caveat.

Furthermore, it is important to acknowledge that the term “lactate clearance” is a physiological misnomer: the formula does not measure true metabolic hepatic clearance, but rather the net change in circulating lactate concentration over time. The term “lactate kinetics” is therefore arguably more appropriate and is used interchangeably in this manuscript.

Finally, the relationship between lactate kinetics and mortality was not linear. A plateau effect was observed beyond certain thresholds of worsening clearance [14], suggesting that lactate kinetics may be more clinically useful when treated as a dichotomous rather than a continuous variable.

### 4.5. Integration with Severity Scores

Both initial lactate and lactate kinetics significantly improved the predictive performance of trauma scores such as RTS, ISS, TRISS, and MGAP. While the individual prognostic value of lactate in trauma is well-established, a key contribution of the present study is the systematic evaluation of the incremental value of lactate kinetics when combined with multiple validated trauma severity scores within a single cohort—an approach that has been seldom conducted comprehensively in the trauma literature. These findings therefore contribute to the evidence base supporting the integration of metabolic markers into multimodal risk stratification frameworks. No significant improvement was observed when lactate was added to SAPS II, likely due to the overlap in physiological information between these variables.

### 4.6. Prediction of Morbidity

Lactate levels and lactate clearance were modest predictors of severe trauma and massive hemorrhage, consistent with previous reports [21,22]. However, their performance remained inferior to clinical and composite scores. In contrast, neither lactate levels nor lactate clearance predicted nosocomial infections in our cohort, which differs from some literature reports and suggests that infection risk is likely influenced by factors unrelated to early metabolic status, such as duration of mechanical ventilation or invasive procedures.

### 4.7. Prognostic Score Development

The composite prognostic score demonstrated good discrimination for in-hospital mortality. Bootstrap internal validation (1000 resamples) yielded an optimism-adjusted AUC of 0.82 (apparent AUC = 0.84), confirming the robustness of the model’s discriminative ability within this cohort while acknowledging a modest degree of shrinkage. The risk of overfitting cannot be entirely excluded given the sample size relative to the number of predictors. The score should therefore be considered a hypothesis-generating tool, and prospective multicenter external validation is strongly encouraged before any clinical application.

### 4.8. Study Limitations

This study has several important limitations. Its single-center retrospective design limits generalizability and precludes causal inference. Selection bias may have been introduced by the exclusion of patients with missing data, who appeared to be more severely ill based on available records. Lactate measurements were obtained after ICU admission, at which point resuscitation had generally been initiated, potentially attenuating the prognostic signal of admission lactate. The analysis of LC H0–H12 is subject to survivorship bias, as documented in Section Characteristics of Patients Who Died Before 12 Hours, which likely results in an overestimation of its apparent predictive performance. Finally, while standardized resuscitation protocols were applied throughout the study period, the retrospective design does not allow complete control for inter-individual variation in therapeutic management.

## 5. Conclusions

Serial lactate monitoring and lactate kinetics analysis represent valuable components of prognostic assessment in severe trauma. Lactate clearance measured between admission and 12 h demonstrated the strongest predictive performance among the lactate parameters evaluated; however, its interpretation must account for the inherent survivorship bias introduced by the exclusion of patients dying within the first 12 h, which likely overestimates its true discriminative ability in an unselected trauma population. Rather than serving as a standalone resuscitation target, lactate kinetics should be integrated within a comprehensive multimodal assessment that incorporates clinical, hemodynamic, and biological parameters. The composite prognostic score proposed in this study demonstrated satisfactory internal validity (optimism-adjusted AUC = 0.82), but requires prospective external validation before routine clinical implementation.

## Figures and Tables

**Table 2:** ROC curve characteristics of lactate levels and lactate clearance (LC) for predicting early mortality (≤48 h) and in-hospital mortality, with 95% confidence intervals.

Parameter	AUC Early	95% CI Early	*p*	AUC In-Hosp.	95% CI In-Hosp.	*p*
LH0 (mmol/L)	0.75	[0.64; 0.85]	<0.001	0.71	[0.65; 0.77]	<0.001
LH4 (mmol/L)	0.74	[0.62; 0.86]	<0.001	0.72	[0.66; 0.78]	<0.001
LH12 (mmol/L)	0.73	[0.59; 0.87]	<0.001	0.73	[0.67; 0.79]	<0.001
LC H0–H4 (%/h)	0.73	[0.63; 0.83]	<0.001	0.70	[0.64; 0.76]	<0.001
LC H0–H12 (%/h)	0.80	[0.71; 0.89]	<0.001	0.75	[0.69; 0.81]	<0.001
LC H4–H12 (%/h)	0.72	[0.59; 0.85]	0.001	0.71	[0.65; 0.77]	<0.001

**Table 3:** Effects of Adding Lactate LH0 and Lactate Clearance LC H0–H12 to RTS, ISS, TRISS, MGAP, SAPS II and in Predicting Mortality.

Variable	OR [95% CI]	*p*
Model 1 (AUC = 0.793): - RTS (per 1-point decrease) - LH0 (per 1 mmol increase) - LC H0–H12 (per 5 %/h increase)		
	1.58 [1.27; 1.98]	<0.001
	1.18 [1.06; 1.31]	0.002
	1.18 [1.10; 1.27]	<0.001
Model 2 (AUC = 0.752) - ISS (per 1-point increase) - Lactates at H0 (per 1 mmol increase) - LC H0–H12 (per 5%/h increase)		
	1.03 [1.01; 1.06]	0.006
	1.14 [1.02; 1.27]	0.013
	1.18 [1.1; 1.27]	<0.001
Model 3 (AUC = 0.797): - TRISS (per 1-point increase) - Lactates at H0 (per 1 mmol increase) - LC H0–H12 (per 5%/h increase)		
	1.02 [1.01; 1.03]	<0.001
	1.13 [1.02; 1.27]	0.019
	1.17 [1.09; 1.25]	<0.001
Model 4 (AUC = 0.817): - MGAP (per 1-point decrease) - Lactates at H0 (per 1 mmol increase) - LC H0–H12 (per 5%/h increase)		
	1.18 [1.11; 1.27]	<0.001
	1.16 [1.04; 1.30]	0.007
	1.18 [1.10; 1.27]	<0.001
Model 5 (AUC = 0.814) - SAPS II (per 1-point increase) - Lactates at H0 (per 1 mmol increase) - LC H0–H12 (per 5%/h increase)		
	1.07 [1.04; 1.1]	<0.001
	1.05 [0.93; 1.18]	0.375
	1.14 [1.07; 1.23]	<0.001

**Table 4:** Independent Predictive Factors of In-Hospital Mortality in Multivariate Analysis.

Parameter	Adjusted OR	[95%] CI	*p*
Age > 60 years	4.10	[1.79; 9.39]	0.001
GCS ≤ 7	5.03	[2.62; 9.67]	<0.001
Prothrombin Time (PT) ≤ 55%	3.38	[1.69; 6.76]	0.001
pH ≤ 7.29	2.07	[1.03; 4.15]	0.041
LC H0–H12 > −2.93	5.55	[2.84; 10.84]	<0.001

**Table 5:** Parameters of a predictive score for in-hospital mortality, including CL H0-H12.

Parameter	OR	Weighting	Adjusted Weighting (on 10-Point Scale)
LC H0–H12 > −2.93	5.55	55	2.75
GCS ≤ 7	5.03	50	2.5
Age > 60 years	4.10	40	2
PT ≤ 55%	3.38	35	1.75
pH ≤ 7.29	2.07	20	1

## Data Availability

The data supporting the findings of this study are available from Center of Traumatology and Major Burns (CTMB); however, restrictions apply due to patient confidentiality and institutional regulations, and therefore, the data are not publicly available. However, the authors have data available upon reasonable request, provided prior approval is obtained from the CTMB Ethical Committee.

## Abbreviations

The following abbreviations are used in this manuscript:

ALAT	Alanine Aminotransferase
ASA	American Society of Anesthesiologists
ASAT	Aspartate Aminotransferase
AUC	Area Under the Curve
CI	Confidence Interval
CTMB	Center of Traumatology and Major Burns
GCS	Glasgow Coma Scale
WBC	White Blood Cells
H0/H4/H12	Time points at admission/4 h/12 h
Hb	Hemoglobin
HCO_3_^−^	Bicarbonate
Ht	Hematocrit
ICU	Intensive Care Unit
IQR	Interquartile Range
ISS	Injury Severity Score
LC	Lactate Clearance (Lactate Kinetics)
MGAP	Mechanism, Glasgow Coma Scale, Age, and Arterial Pressure score
OR	Odds Ratio
PaCO_2_	Partial Pressure of Carbon Dioxide
Plq	Platelets
PT	Prothrombin Time
RBC	Red Blood Cells
ROC	Receiver Operating Characteristic
RTS	Revised Trauma Score
RTA	Road Traffic Accident
SAPS II	Simplified Acute Physiology Score II
TRISS	Trauma and Injury Severity Score
